# Relevant methane emission to the atmosphere from a geological gas manifestation

**DOI:** 10.1038/s41598-021-83369-9

**Published:** 2021-02-18

**Authors:** Adriano Mazzini, Alessandra Sciarra, Giuseppe Etiope, Pankaj Sadavarte, Sander Houweling, Sudhanshu Pandey, Alwi Husein

**Affiliations:** 1 Centre for Earth Evolution and Dynamics (CEED), University of Oslo, Oslo, Norway; 2 Istituto Nazionale di Geofisica e Vulcanologia, via di Vigna Murata 605, 00143 Rome, Italy; 3 Faculty of Environmental Science and Engineering, Babes Bolyai University, Cluj-Napoca, Romania; 4 SRON Netherlands Institute for Space Research, Earth Science Group (ESG), Utrecht, The Netherlands; 5 Department of Climate, Air and Sustainability, TNO, Utrecht, The Netherlands; 6 Department of Earth Sciences, Vrije Universiteit, Amsterdam, The Netherlands; 7 Pusat Pengendalian Lumpur Sidoarjo (PPLS), Suarabaya, Indonesia

**Keywords:** Geochemistry, Environmental impact

## Abstract

Quantifying natural geological sources of methane (CH_4_) allows to improve the assessment of anthropogenic emissions to the atmosphere from fossil fuel industries. The global CH_4_ flux of geological gas is, however, an object of debate. Recent fossil (^14^C-free) CH_4_ measurements in preindustrial-era ice cores suggest very low global geological emissions (~ 1.6 Tg year^−1^), implying a larger fossil fuel industry source. This is however in contrast with previously published bottom-up and top-down geo-emission estimates (~ 45 Tg year^−1^) and even regional-scale emissions of ~ 1–2 Tg year^−1^. Here we report on significant geological CH_4_ emissions from the Lusi hydrothermal system (Indonesia), measured by ground-based and satellite (TROPOMI) techniques. Both techniques indicate a total CH_4_ output of ~ 0.1 Tg year^−1^, equivalent to the minimum value of global geo-emission derived by ice core ^14^CH_4_ estimates. Our results are consistent with the order of magnitude of the emission factors of large seeps used in global bottom-up estimates, and endorse a substantial contribution from natural Earth’s CH_4_ degassing. The preindustrial ice core assessments of geological CH_4_ release may be underestimated and require further study. Satellite measurements can help to test geological CH_4_ emission factors and explain the gap between the contrasting estimates.

## Introduction

Methane (CH_4_) is a greenhouse gas 28 times more powerful than carbon dioxide (CO_2_) on a 100 year time horizon^1^. It is released to the atmosphere by both natural and anthropogenic sources, with a global emission of ~ 560 teragrams per year (Tg year^−1^)^2,3^. About 30% of this methane is fossil^4^, characterized by the absence of radiocarbon (^14^C) that is present in modern biological gas. Fossil CH_4_ is mostly released by fugitive emissions during oil and gas extraction and distribution, and coal mining (about 100–145 Tg year^−1^^3,5^). In addition, CH_4_ is also naturally emitted through the Earth’s degassing via surface gas manifestations (seeps, mud volcanoes, diffuse microseepage) in petroleum-bearing sedimentary basins and geothermal areas (e.g.^6^). Understanding the strength of this geological source provides better constraints on the anthropogenic fossil fuel fraction^5,7^. Bottom-up emission estimates (based on inventories and measurements of emission factors) and top-down estimates (atmospheric and ice core data and inverse modelling) converge to a global geo-CH_4_ output of around 45 Tg year^−1^ (from 27 to 70 Tg year^−1^) (e.g.^8^). In contrast, recent estimates based on preindustrial-era ice core ^14^CH_4_ measurements suggest values one to two orders of magnitude lower (from 0.1 to 5.4 Tg year^−1^, with a median value of 1.6 Tg year^−1^)^7^, which would greatly increase the estimate of the anthropogenic fossil fuel fraction. Hmiel et al.^7^ based their study on the measurements of the amount of fossil CH_4_ present in the air trapped in preindustrial ice cores, and thus before the influence of hydrocarbon exploration and production. The CH_4_ contribution from the fossil fuel industry was inferred by assuming that the measured ice-core CH_4_ represents the natural geological emissions, and also expecting that this natural degassing remained relatively constant over the last few centuries.

In short, there is a large discrepancy between the geological CH_4_ source estimates by Hmiel et al.^7^ and those previously proposed by several scholars for specific types of geological sources, e.g. mud volcanoes^6,9,10^; microseepage^11,12^; and submarine seeps^8,13,14^. This disparity appears even more striking when considering local and regional geo-CH_4_ emission estimates. For example seeps in Alaska have an estimated CH_4_ release of 0.7–1.4 Tg year^−1^^15^, while submarine seeps in the East Siberian Arctic Shelf have a total CH_4_ output to the atmosphere of 3 Tg year^−1^^16^. The Hmiel et al.^7^ estimate is inconsistent with field flux measurements, since it would imply seepage emission factors one to two orders of magnitude lower than those assessed so far (for example, about 3000–6000 tonnes km^−2^ year^−1^ for mud volcanoes^6^).

**Figure 1:**
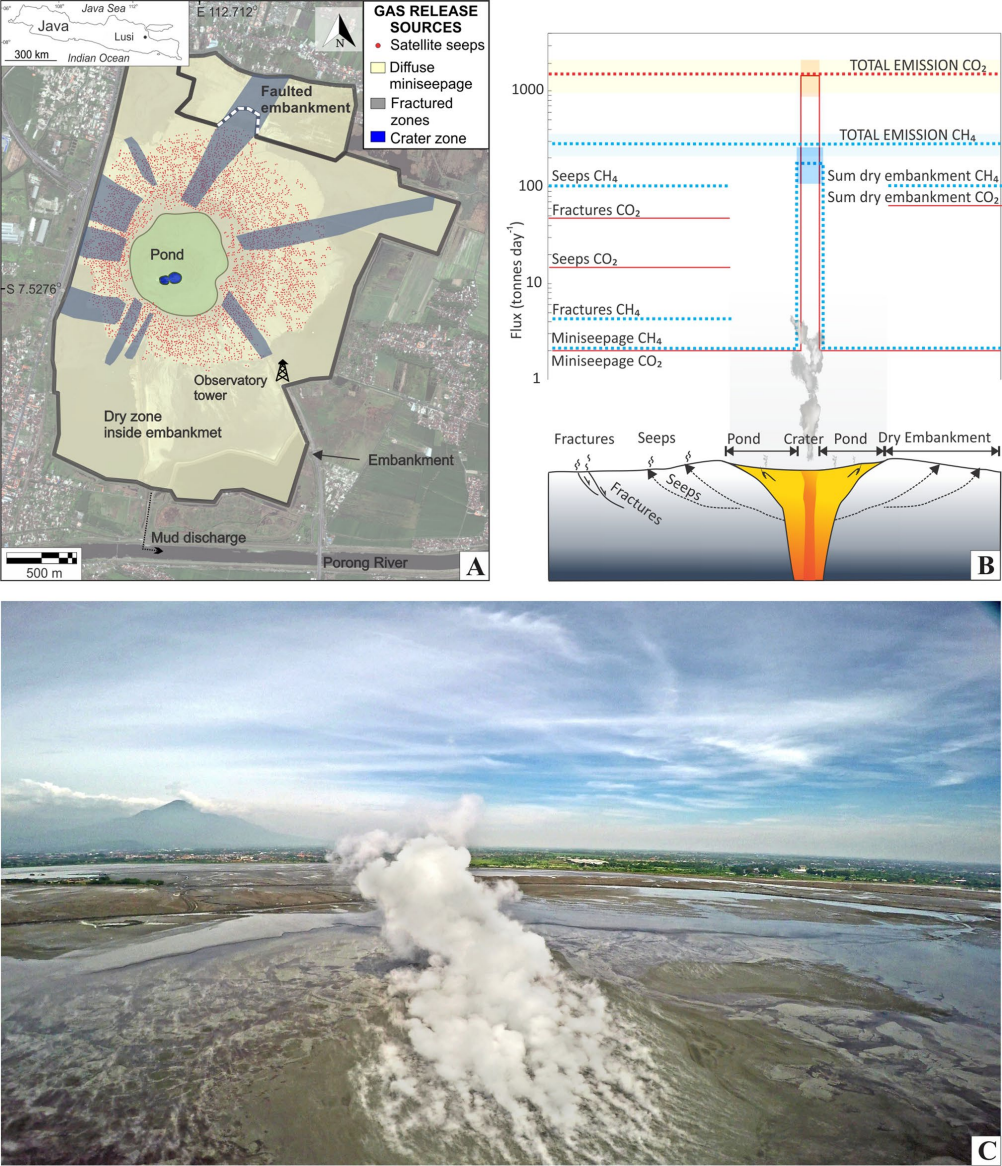
Gas emissions at Lusi site. (**A**) High resolution Ikonos satellite image of Lusi area in August 2014; Additional details, exported from basemap obtained from Esri ArcGIS ArcMap 10.2.1 and overlaid using Corel Draw X7, indicate the main features and the gas release sources identified in the region. Inset map of Java Island with indicated Lusi location. (**B**) Detailed outputs of gas emissions from the different sources around the eruption site. (**C**) Drone view of the plume during its regular geysering activity. The second vent is behind the main one and the Arjuno-Welirang volcanic complex in the background. The inaccessible 650 m in diameter pond surrounding the crater zone is filled by hot mud and laterally extensive oil slicks.

Here, we report CH_4_ emissions from a single, large gas manifestation whose flux is already within the lower range of the global estimate derived by ice core ^14^CH_4_ measurements. For the first time, satellite (TROPOMI) observations are used to test and support ground-based flux measurements. The degassing site is known as Lusi (‘LUmpur’ -meaning mud in Indonesian, and ‘SIdoarjo’ -the district name), located in the northeast Java Island, Indonesia^17^. Lusi is a hybrid Sediment-Hosted Geothermal System (SHGS)^18,19^ triggered after the 27th May 2006 Java earthquake^20^. Numerous mud bursting vents appeared on the 29th of May 2006, almost two days after a 6.3 M earthquake struck the island. These eruption sites developed over a distance of > 1 km along a system of fractures that follows the orientation of the Watukosek fault system^20^. This is a NE-SW regional tectonic discontinuity running from the Arjuno-Welirang (AW) volcanic arc towards the NE of Java, intersecting Lusi and several mud volcanoes (e.g.^21–23^). Within weeks after the inception, the active eruption sites expanded in size, with the largest one developing a 100 m wide vent releasing boiling water, mud, rock clasts, oil and gas. Up to 180,000 m^3^ day^−1^ of mud were expelled, resulting in submerged villages and displacement of more than 60,000 people^20,24^. Two major vents currently erupt in the crater zone, alternating between periods of regular activity and powerful geysering events, making Lusi a clastic-dominated geysering-like system^25^. These two Lusi vents are isolated inside a ~ 650 m diameter circular pond of fluidized mud, framed by a vast area of dry mud breccia hosting thousands of bubbling seeps. The region covered with mud breccia spans over ~ 7 km^2^ and is confined by a tall embankment built to prevent flooding expansion (Fig. 1A).

Central vents at Lusi primarily discharge aqueous vapour (~ 98 vol.%) belonging to the hydrothermal component of the system. In addition, CH_4_ and CO_2,_ are released at variable concentrations both from the boiling vents, satellite peripheral seeps, and fractured ground^18,23,26^. Converging geophysical, geochemical, petrography, and modelling data indicate that magmatic/hydrothermal CO_2_-rich fluids, migrating from the neighbouring AW volcanic complex, flushed through the hydrocarbon-rich back-arc sedimentary basin triggering the formation of over-pressurised gas pools^18,27–32^.

**Figure 2:**
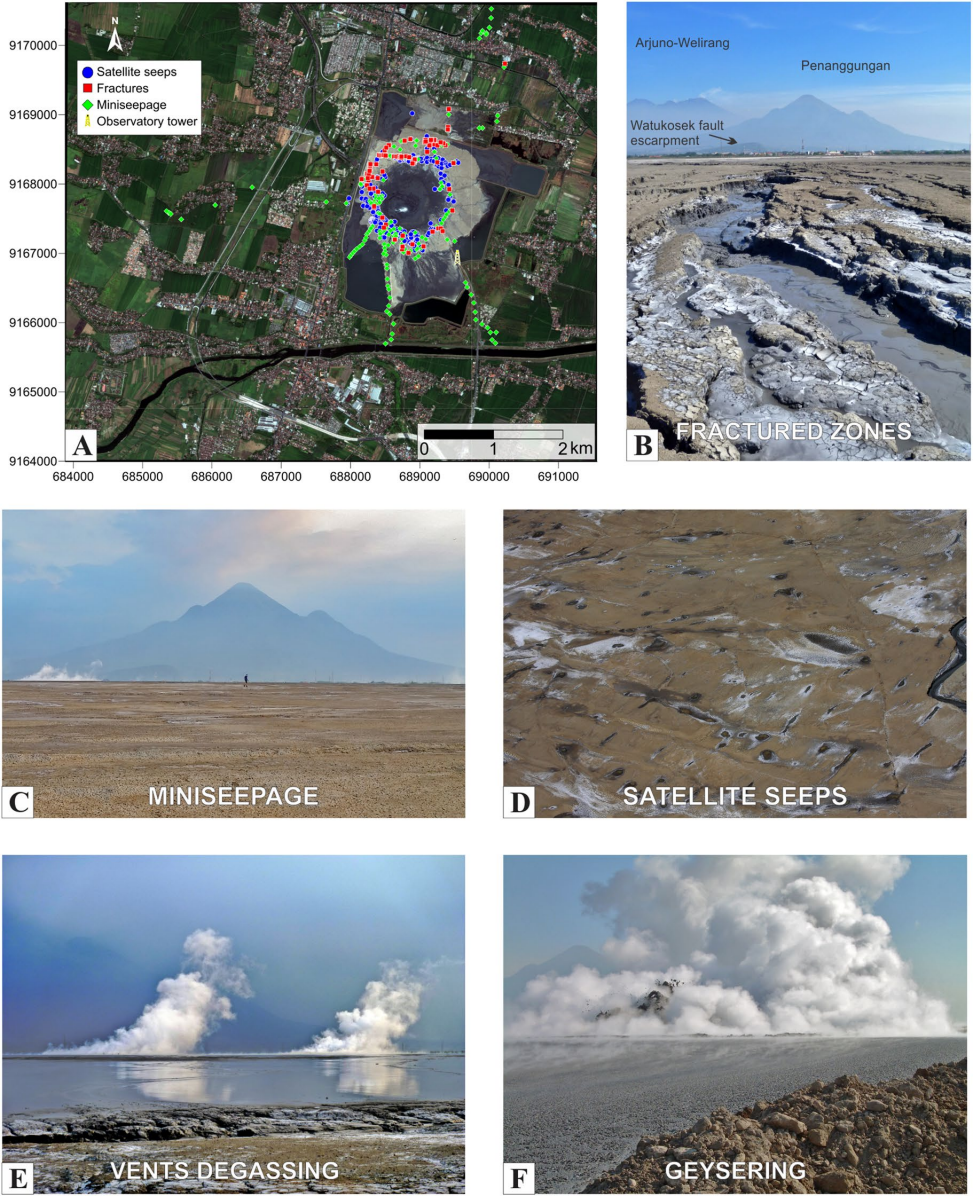
Measurements and identified gas release sources at Lusi. (**A**) High resolution Ikonos satellite image of Lusi area in August 2014 with additional overlaid basemap constructed using Esri ArcGIS ArcMap 10.2.1. Symbols indicate the positions, and the equivalent degassing modes, of the flow measurements done with accumulation chamber. (**B**–**E**) The four main degassing modes identified in this study: (**B**) fractured zone extending in NE–SW direction towards the volcanic complex in the background; (**C**) field view of the vast region where miniseepage occurs in undisturbed surface; (**D**) aerial view of dozens of satellite seeps (for scale the stream on the right side is ~ 1 m wide); (**E**) the two active vents during regular activity, view from the edge of the not accessible pond. (**F**) Example of geysering activity.

**Figure 3:**
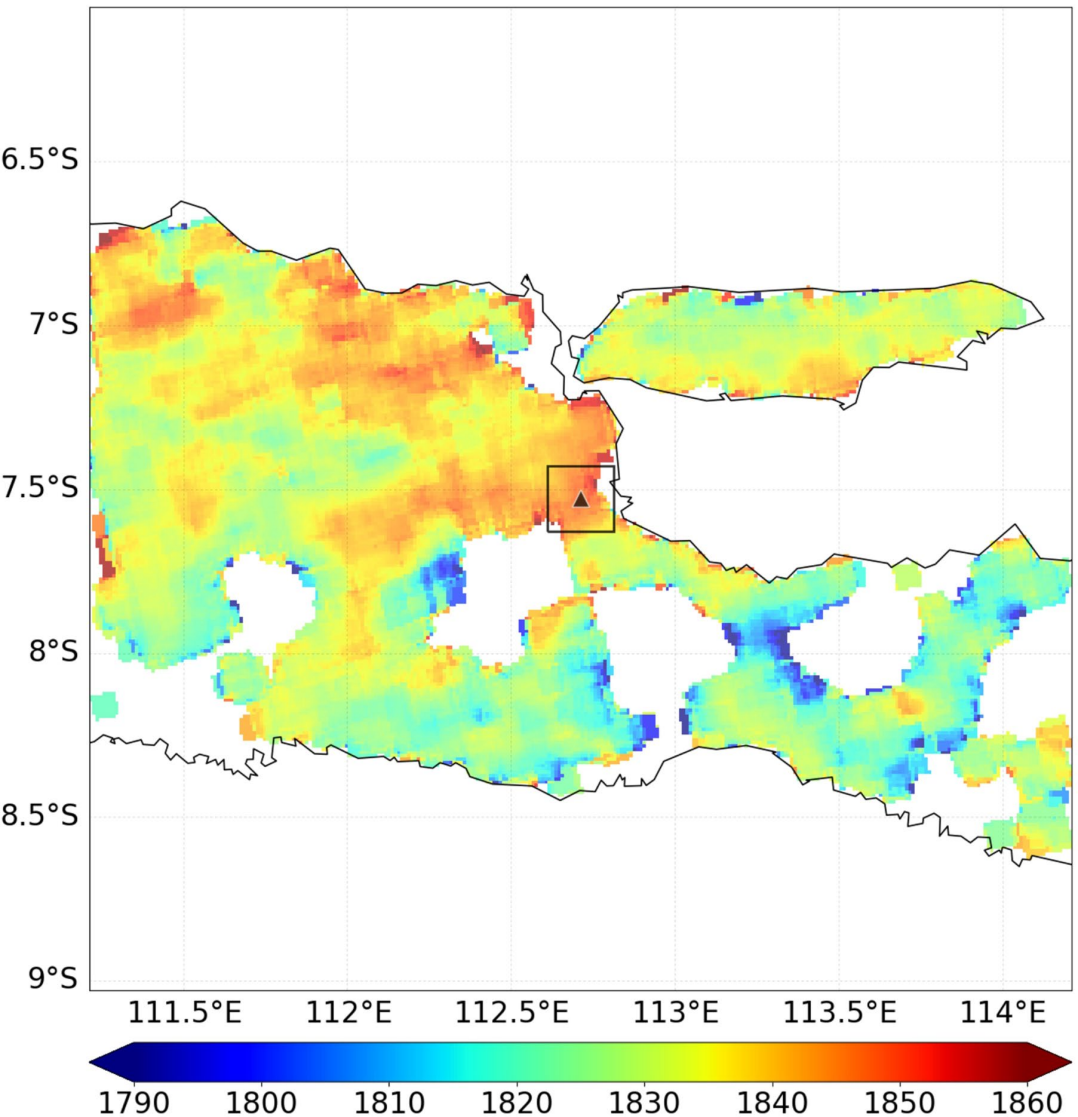
An oversampled (0.01° × 0.01°) map of TROPOMI data averaged over the study domain from May 2018 till July 2019. The location of Lusi is indicated by a triangle and the square denotes the source box. Units in ppb. Map generated using Python 2.7.13 version.

Here, we estimate the total CH_4_ emissions from Lusi using both ground-based and satellite (TROPOMI) measurements (Figs. 1, 2, 3). CO_2_ emission is additionally measured by ground-based techniques. Ground-based and remote sensing methods provide very similar estimates, confirming the order of magnitude of the emission factors of large seeps used in global bottom-up emission estimates^6^.

## Results

### Gas emission structures and related fluxes

We studied and classified the Lusi degassing modes identified throughout the region inside the embankment and performed CH_4_ and CO_2_ flux measurements in all of them (Figs. 1, 2). We recognised that, in addition to two main *central vents* (active in what we define as the crater zone), the gas exhales abundantly from three different emission modes: (a) diffuse invisible seepage (*miniseepage*) occurring throughout the area covered by mud, (b) degassing through a network of *fractures,* parallel and antithetic to the Watukosek fault system, and (c) thousands of *satellite seeps* scattered around the crater zone inside the embankment area (see details in “Supplementary Material”, Fig. 2). For each of these degassing modes we applied specific ground-based flux measurement approaches, including the closed-chamber method (655 flux measurements), crater plume monitoring and volume measurements (205 measurements), and up-scaling techniques following widely used methods in geological gas emission studies (“Methods” and “Supplementary Material”).

Table 1 and Fig. 1B show the CH_4_ and CO_2_ fluxes released to the atmosphere from the four Lusi degassing modes (i.e. crater zone, miniseepage, fractured zones and satellite seeps). Details of flux calculations are provided in the “Supplementary Material”, Figs. S1, S2, S3 and S4. The main vent, whose gas composition is characterized by an average CO_2_/CH_4_ ratio ~ 3^18,26^, releases in total ~ 42 (from 26 to 61) ktonnes CH_4_ year^−1^, and ~ 340 (from 213 to 496) ktonnes CO_2_ year^−1^. The gas flux from the second vent was not measured, but long-term observations and video records show that its activity is similar to that of the main vent with a focussed plume of ~ 35 m in diameter. Using the same approach applied for the main vent, we calculate a mean emission of ~ 22 ktonnes CH_4_ year^−1^, and ~ 176 ktonnes CO_2_ year^−1^. Details for crater zone emission estimates are provided in the “Supplementary Material”.

**Table 1:** CH_4_ and CO_2_ fluxes released to the atmosphere from the four Lusi degassing systems (two vents in the crater zone, miniseepage, fractured zones and satellite seeps) estimated by ground-based measurements.

Seepage mode	N. data	ϕCH_4_ (tonnes year^−1^)	ϕCO_2_ (tonnes year^−1^)	ϕCH_4_ (tonnes km^−2^ year^−1^)	ϕCO_2_ (tonnes km^−2^ year^−1^)	Area (km^2^)
Vent1 LOW		26,350	212,822			
Vent1 MEAN	205^a^	42,040	339,551			
Vent1 MAX		61,372	495,690			
Vent2 LOW		13,660	110,327			
Vent2 MEAN		21,794	176,023			
Vent2 MAX		31,815	256,965			
Miniseepage LOW		346	471	59	80	
Miniseepage MEAN	175	774	734	131	124	5.91
Miniseepage MAX		2047	1752	346	296	
Fractured zones LOW		15	5595	12	4176	
Fractured zones MEAN	129	1573	17,484	1174	13,047	1.34
Fractured zones MAX		7022	36,586	5240	27,303	
Satellite seeps	351	35,960	5325			
Total excluding crater zone		38,307	23,543			
Total LOW		78,316	346,692	10,802	47,820	
Total MEAN		102,141	539,117	14,088	74,361	7.25
Total MAX		131,494	776,198	18,137	107,062	
TROPOMI LOW		53,000				
TROPOMI MEAN		140,000				
TROPOMI MAX		227,000				

^a^Number of vent plume measurements; ϕ = estimated gas flux. The accuracy of the flux measurements completed with the portable fluximeter inside the embankment zone (miniseepage, fractures zones and satellite seeps) is ± 10%.

The surrounding area, including fractures, satellite seeps and diffuse degassing (miniseepage) over ~ 7 km^2^, releases ~ 38 ktonnes CH_4_ year^−1^, and ~ 24 ktonnes CO_2_ year^−1^. Ground-based flux measurements suggest, therefore, a total CH_4_ emission of around 0.1 Tg year^−1^ (78.3–131.5 ktonnes year^−1^) (Fig. 1B, Table 1).

The amount of CH_4_ released from the crater zone and that from the surrounding degassing are of the same order of magnitude. CO_2_ instead is mostly released from the vents, representing the core of the hydrothermal manifestation as indicated by hot fluids and abundant water vapour. It has been suggested that the CO_2_-dominated gas released from the vents results from the rapid de-pressurization of the fast-rising fluids combined with the exsolution of the dissolved pore water gas ongoing at high temperatures (i.e. > 100 °C) at near surface conditions^18,20^. The gas from the satellite seeps is instead CH_4_-enriched. The degassing in the surrounding zone is likely related to colder peripheral pathways that branch off from the main conduit in combination with release of gas stored within the thick deposits of mud breccia^18^. These deposits range in thickness from hundreds to tens of metres with increasing distance from the central area^33,34^.

We stress that the gas emission estimates are conservative. They do not include the continuous degassing in the 650 m diameter circular pond framing the crater zone. In this inaccessible area, we assigned a low averaged soil miniseepage value, however aerial images show intense and ongoing diffuse gas bubbling activity. In addition, the crater zone emission was calculated during Lusi steady state degassing (i.e. regular activity). Importantly, our budget does not include the powerful geysering activity that characterizes Lusi for about fifty percent of the time^25^. Ultimately, elevated air content estimates within the plume volume (i.e. 75%) were considered in our calculations, although video records often showed an apparently more homogenously dense gas plume.

Figure S4C shows the monitoring of Lusi mud flow rate since the inception in May 2006. Between 2014 and 2016 (i.e. survey time described herein) Lusi had the lowest recorded flow rate. Previous studies revealed that a direct correlation exists between the increase in flow rate and the gas released from the plume^25,35,36^; therefore our conservative degassing estimates can safely be applied throughout Lusi’s activity. Daily observations, videos, and monitoring show that during the first years Lusi had long-lasting geysering phases and higher mud flow rates (i.e. up to 180,000 m^3^ day^−1^), and developed a system of vigorous satellite seeps extending for kilometres around the main crater zone. Therefore, much greater degassing is expected to have occurred between 2006 and 2011, and from the end of 2015 until the end of 2018. Interpolating our flow rate measurements (Fig. S4C), we calculate that between May 2006 and January 2019 Lusi discharged a total of ~ 0.3 km^3^ of mud breccia.

### TROPOMI satellite measurements

We obtained an independent emission estimate from TROPOMI retrievals of total column CH_4_ (XCH_4_) collected over eastern Java between May 2018 and July 2019 (Fig. 3). XCH_4_ enhancements are found in the Surabaya and Sidoarjo region, including the location of Lusi, on the order of 8–9 ppb. We estimated the CH_4_ emissions from Lusi of 140 ± 87 ktonnes year^−1^ using the mass balance approach (see “Methods”). Figure 1A,B highlight the presence of many smaller (fractured zone, mini seepage, satellite seeps) and larger (crater zone) methane emitting seepage modes present throughout the area considered. Similarly, Table 1 shows that the crater (situated in the center) emits around 40–93 ktonnes year^−1^ methane while satellite seeps, which are uniformly spread across the whole area accounts for a total of 36 ktonnes year^−1^, with a smaller fraction from other seepage mode (0.5–9 ktonnes year^−1^). Given this variety in methane fluxes, we expect to detect diffuse XCH_4_ enhancements rather than distinct plumes in TROPOMI. The higher estimated emissions from TROPOMI are consistent with a higher mud flow rate measured during 2018–2019 compared to that measured during the 2014–2016 ground-based survey period. However, the relatively high TROPOMI estimates are not linearly related to the increase in mud flow rate observed for the in-situ measurements (Fig. S4C). The low mean estimate from TROPOMI can be due to overestimation of anthropogenic emissions in EDGAR (see more details in “Supplementary Material”). Also, the low wind speeds observed in the region introduces uncertainty (tabulated in Table S1 and Fig. S6A). The larger uncertainty bound of the satellite-derived emissions can accommodate a source rate > 200 ktonnes year^−1^, which is significantly larger than the ground-based estimation. The high uncertainty in the TROPOMI-derived estimate is mainly due to sensitivity to the choice of the background and source box, as well as the difficulty to unequivocally distinguish Lusi emissions from others anthropogenic sources in the region. The anthropogenic emissions and wetland emissions are estimated to vary respectively by 12–47% and 0.04–0.4% of the total emission in source boxes varying in size between 0.2° × 0.2° and 0.7° × 0.7° in intervals of 0.1° (Fig. S5). The contribution of the surface emissions is relatively small for the smallest source box, but we cannot exclude the possibility that the local XCH_4_ enhancements are influenced by surrounding emissions, particularly given the generally low wind-speeds (< 2 m s^−1^). It is interesting to note that, aside from rare exceptions, the predominant winds throughout the monitored timeframe have easterly and south-easterly directions (Fig. S6B). No obvious methane signal can be detected to the south of Lusi despite the fact that the settlement distributions are similar to both the northern and southern side of the eruption site. This observation strengthens the fact that the TROPOMI estimates are consistent with a sizeable source from Lusi for any choice of region (Fig. S6E).

## Discussion

Although Lusi is neither a pure hydrothermal system nor a magmatic volcano, its CO_2_ output of ~ 0.35–0.78 Tg year^−1^ is within the range (0.006–19 Tg year^−1^) of volcanic emissions, and comparable with the emissions of Erebus (Antarctica), White Island (New Zealand) and Augustine (USA) volcanoes (an updated list of volcanic CO_2_ output are reported by^37^ and Refs. therein). Large tectonic and hydrothermal/geothermal systems release CO_2_ amounts similar to those measured at Lusi^37,38^. Therefore, as a geological CO_2_ source, Lusi is not an exceptional case. Global volcanic CO_2_ emissions (~ 600 Tg year^−1^^37,39^) represent a very modest component of the atmospheric carbon budget, two and three orders of magnitude lower than anthropogenic and natural sources, respectively^2^.

Conversely, natural geological emissions appear to be a significant fraction of the atmospheric CH_4_ budget (~ 45 Tg year^−1^^8^), roughly accounting 8% of total emissions. Recent analyses, based on preindustrial-era ice core ^14^CH_4_ measurements, suggest instead a much lower global output, ranging from 0.1 to 5.4 Tg year^−1^^7^. These estimates diverge also with the values reported herein, which reveal that the Lusi emissions alone already match the minimum range assessed by Hmiel et al.^7^  for the entire planet. Moreover, in a global onshore seep inventory, 76 seeps were identified as “big emitters”, i.e. potentially releasing methane in the order of 10^4^ tonnes year^−1^^6^. This list includes Lusi. The emission estimate in the seep inventory is based on statistically derived emission factors and seepage area calculated by image analysis (see details in^6^). An overall (vents plus diffuse seepage) emission factor of 7.1 ktonnes km^−2^ year^−1^ (statistically derived^6^) was applied to Lusi, resulting in a predicted potential release of about 50 ktonnes CH_4_ year^−1^. This is equivalent to the lower estimate by TROPOMI, or less than half the mean value estimated by either TROPOMI or ground-based techniques, which indicates an overall emission factor exceeding 14 ktonnes km^−2^ year^−1^ (~ 5.3 ktonnes km^−2^ year^−1^ excluding the crater zone). We can hypothesize that numerous of the continuously and actively degassing mud volcanoes and similar seep systems worldwide, which differently from Lusi are dominated by CH_4_ and not CO_2_, may have CH_4_ emission factors at least of the same order of magnitude of that estimated in this work. For example, the “big emitters” inventory^6^ includes 27 mud volcanoes or mud volcano clusters, mostly in Azerbaijan, with a size similar or exceeding that from Lusi. These emitters may then release CH_4_ amounts of the same order of magnitude of those degassed by Lusi in the peripheral part through invisible miniseepage, fractured zones and active seeps. Such seepage potential suggests that the ice core ^14^CH_4_ study^7^ may have underestimated the natural Earth’s CH_4_ degassing. In any case, the discrepancy existing between the field plus satellite measurements and the ice core ^14^CH_4_ estimates implies that the latter requires further investigations and evaluations.

The abundant methane release measured in the accessible area around Lusi’s crater (i.e. excluding the crater zone) is comparable to that of major leaks related to fossil fuel industry, such as gas compressors in Turkmenistan, recently detected by the TROPOMI and GHG-Sat D satellites (about 129 ktonnes year^−1^^40^), and it is higher than the largest reported methane point sources, coal mines and landfills, in the United States (10–100 ktonnes year^−1^^41^).

Our results reveal that satellite-derived emission estimates are becoming a fundamental tool to validate ground-based flux measurements. Refined remote-sensing estimates may be a valid substitute for field activities that are time-consuming and often impossible to be completed in dangerous or inaccessible regions. Identification and detection of geo-methane emission points/areas, including large active seeps, vents and mud volcanoes, using satellite observations will be an essential challenge to improve global estimates of Earth's methane degassing and, indirectly, quantify the anthropogenic emission from fossil fuel industries.

## Methods

This works combines the observations and measurements conducted during daily routine monitoring of the Indonesian Ministry Agencies (BPLS-PPLS) and the data collected during more than a dozen of dedicated fieldworks conducted since the beginning of the Lusi eruption in 2006. Gas flux measurements were designed and performed according to the several degassing modes (crater zone, miniseepage, fractured zones and satellite seeps, Fig. S1) described in detail in the “Supplementary Material”.

CH_4_ and CO_2_ flux from the main vent was estimated by measuring gas plume vertical velocity (period 2015–2019), through a theodolite (Topcon AT-G2) mounted on the observatory tower located at the south-eastern part of Lusi embankment, knowing gas plume density (based on H_2_O, CO_2_ and CH_4_ relative composition) and CH_4_ and CO_2_ concentration/ratio (from^18,26^). For the procedures and data elaboration, see “Supplementary Material”.

Gas flux from the miniseepage, fractured zones and satellite seeps was measured by closed-chamber technique. The system used a 20 cm-diameter metallic box connected to a West System sensor package (Pontedera, Italy) including a laser CH_4_ sensor (Tunable Diode Laser Adsorption detector, precision and lower detection limit of 0.1 ppmv) and an infrared CO_2_ detector (LICOR–LI820, accuracy of 2% and repeatability is ± 5 ppmv). The flux is derived by measuring the concentration build-up within the box over time (e.g.^23^).

The sample site selection was mainly linked to the site logistics and the presence of dry walkable mud inside the embankment. In this area, a total of 655 CO_2_ and CH_4_ flux measurements were performed (during November–December 2014, June 2015 and May 2016) from the three degassing modes (Fig. 2A).

In total, 351 satellite seeps with varying amount of water content, microbial activity and extension, were measured. The flux measurements were also carried out radially from individual seeps to assess the extension of the macro-seepage area excluding miniseepage. This datum, estimated to be ~ 2 m, was important for the output estimation since it takes into account the area of the individual seeps.

In total, 129 flow measurements were carried out both along the raised edge and in the depressions present in the central part of the main fractured/faulted zones oriented NE–SW (i.e. Watukosek strike slip fault system) or through the antithetic equivalents (Siring fault system) (e.g.^21–23^^,^^33^).

In total, 175 flux measurements were carried out on diffuse invisible seepage (miniseepage) throughout the area covered by walkable dry mud, trying to obtain a distribution as homogeneous as possible of the data.

Flux data were elaborated by statistical and spatial analysis software packages (Surfer 12.0, Golden Software, Inc.; Statistica 10.0, StatSoft, Inc.; ArcGIS, ESRI, Inc.) to estimate total gas emissions (“Supplementary Material”). Miniseepage and fractured zone emissions, being areal degassing modes, were estimated by Natural Neighbour interpolation and volume method (Surfer 12.0, Golden Software, Inc.). Total emission from satellite seeps was estimated by summing individual fluxes from measured and modelled seeps (“Supplementary Material”).

Recent studies^42,43^ have shown independent TROPOMI estimates using the mass balance method to quantify methane emissions using XCH_4_ enhancements over an area source relative to an upwind background. The study presented herein uses the mass balance method from Buchwitz et al.^44^ to quantify methane emission using a background region defined based on boundary layer averaged wind speed and direction from the ECMWF ERA5 reanalysis. The contributions from anthropogenic, biomass burning and wetland emissions in the source box are accounted for using the EDGARv5.0^45^, GFED4.1 s^46^ and WetCHARTs version 1.0^47^ emissions. A total of 50 orbits were screened for data availability in the period May 2018–July 2019 requiring > 100 valid retrievals (quality flag q_a_ ≥ 0) over the analyzed domain per orbit. The data from these orbits have been regridded and averaged at a resolution of 0.1° × 0.1°. A negative correlation of − 0.68 was found between the averaged XCH_4_ and aerosol optical thickness (AOT). A linear regression between XCH_4_ and AOT (Fig. S6D) yields a slope of − 364 ppb per unit of AOT, which has also been used to account for the influence of aerosols on the XCH_4_ retrieval as a sensitivity study.

The uncertainty in TROPOMI-inferred emissions was represented by one standard deviation across an ensemble of estimates and in wind speed, and calculated as sum in quadrature. The ensemble of estimates was created by varying the following parameters: (a) background, varied at an interval of 0.25 (times length of source box) till the dimension length equals the length of the source box, (b) the regridding resolution, perform the analysis at different resolution 0.01° × 0.01°, 0.05° × 0.05° and 0.1° × 0.1°, and (c) lastly vary the source box from 0.2° × 0.2° to 0.7° × 0.7° to test the influence of anthropogenic emissions on our estimate for Lusi (shown in Fig. S5). Finally, the uncertainties in wind speed were accounted by considering average winds over the source boxes at different time steps (0600 UTC h ± 2) and the variability in the winds over different source box. The uncertainties (one standard deviation) in wind speed is tabulated in the “Supplementary Material” (Table S1). Further details about the quantification of emission using TROPOMI data can be found in the “Supplementary Material”.

## Supplementary Information

Supplementary Information 1.

## Data Availability

All data generated or analysed during this study are included in this published article and its “Supplementary Material” files. ECMWF ERA-5 reanalysis data is freely available at https://cds.climate.copernicus.eu. TROPOMI data can be accessed at Copernicus Open Access Hub (https://scihub.copernicus.eu/).
