# Supplementary material for: Relevant methane emission to the atmosphere from a geological gas manifestation
Source: Sci Rep. 2021 Feb 18;11:4138. doi: 10.1038/s41598-021-83369-9 (PMC7892996; doi:10.1038/s41598-021-83369-9)
Supplement: Supplementary file 1 — Supplementary Information 1. [file 41598_2021_83369_MOESM1_ESM.pdf]

SUPPLEMENTARY MATERIAL

Relevant methane emission to the atmosphere from a geological gas manifestation

Adriano Mazzini[1], Alessandra Sciarra[2], Giuseppe Etiope[2,3], Pankaj Sadavarte[4,5], Sander Houweling[4,6], Sudhanshu Pandey[4], Alwi Husein[7]

[1]Centre for Earth Evolution and Dynamics (CEED), University of Oslo, Oslo, Norway.

[2]Istituto Nazionale di Geofisica e Vulcanologia, via di Vigna Murata 605, 00143 Rome, Italy

[3]Faculty of Environmental Science and Engineering, Babes Bolyai University, Cluj-Napoca, Romania

[4]SRON Netherlands Institute for Space Research, Earth Science Group (ESG), Utrecht, the Netherlands

[5]Department of Climate, Air and Sustainability, TNO, Utrecht, the Netherlands

[6]Department of Earth Sciences, Vrije Universiteit, Amsterdam, the Netherlands

[7]Pusat Pengendalian Lumpur Sidoarjo (PPLS), Suarabaya, Indonesia

Lusi is a Sediment-Hosted Geothermal System (SHGS). These systems[1], characterized by carbon dioxide (CO_2) of deep geothermal origin (thermometamorphic or mantle-derived), interact with CH_4-rich fluids in shallower sedimentary rocks, leading to a hybrid CH_4-CO_2 surface seepage system. SHGSs often feature muddy craters or bubbling pools and can be confused with, either mud volcanoes (sedimentary volcanism; [2]) or mere geothermal/volcanic emissions. The methane vented at Lusi is mainly thermogenic (δ^{13}C from -40 to -49 ‰), although a possible mixing with shallower microbial gas (resulting in δ^{13}C: -51.8 ‰) was observed in the initial stages of Lusi's activity[3].

1. Degassing modality

Lusi is a complex and multiple degassing system, with different components and modalities of gas release from the ground. The recognition of the degassing components was a prerequisite to design and perform gas flux measurements (Table 1).

The main degassing zone, which is object of the present work, covers an area of ~7 km^2, framed by a 12 m tall embankment (Fig. 1A, 2A). This surface can be divided in three main sectors.

(a) The central part (defined as crater zone) hosts two large active vents. These are characterized by constant gas and mud breccia release resulting in tens of meters tall plumes composed respectively of: 98% aqueous vapour, 1.5% CO_2 and 0.5% CH_4[;3,4].

Drone thermal images showed the presence of two distinct hot zones that correspond precisely to the two active vents, where the most active bubbling occurs releasing deeper and hotter fluids [5]. A significant thermal effect around the main vent extends over a surface of 200 m^2, and about 80 m^2 for the secondary one. The main vent clearly defines a surface temperature pattern of concentric rings. The crater zone has the highest temperatures and is characterized by the constant activity of bursting bubbles that reach the size of several meters.

(b) A ~650 m diameter subcircular pond (>0.3 km^2) surrounds the active vents region. This pond, consisting of hot mud, is not accessible and hosts countless bubbling points, which vary in size from meters to decimetres scale and change position continuously together with the mud breccia flows that radially expand from the crater zone.

(c) The external region consists of dry walkable mud-breccia and is sheared by radial streams that flush the hot mud erupted from the vents. In this area three main degassing types could be identified: soil miniseepage, fractured zones, satellite seeps.

i) *Soil miniseepage*: a diffuse exhalation occurring throughout the walkable area inside the embankment, where no specific features are observed and mud appears to be generally undisturbed (Fig. 1A, 2C). It is analogous to “miniseepage” typically reported around seeps and mud volcanoes (e.g.; [6]).

ii) F*ractured zones:* characterized by ten to hundred meter long fissures, centimetres to several meters wide and up to 3-5 meters deep (Fig. 1A, 2B, S2). They were mapped combining direct

field observations and high resolution satellite images as well as drone photogrammetry. The main fractured/faulted zones are NE-SW oriented (i.e. part of the Watukosek strike slip fault system) or coincide with the system of antithetic fracture zones (Siring fault system)[7-10].

iii) S*atellite seep*s: active bubbling pools, broadly distributed throughout the entire region inside the embankment (Fig. 1A, 2D, S3). Seeps are circular depressions with an average diameter in the order of 20-30 cm, up to 1.5 m, and typically expel gas, water and, in some rare instances, mud, thus resulting in the formation of small gryphons. These seeps are part of an intricate subsurface plumbing system that continuously mutates in time as the fluids follow preferential pathways to reach the surface.

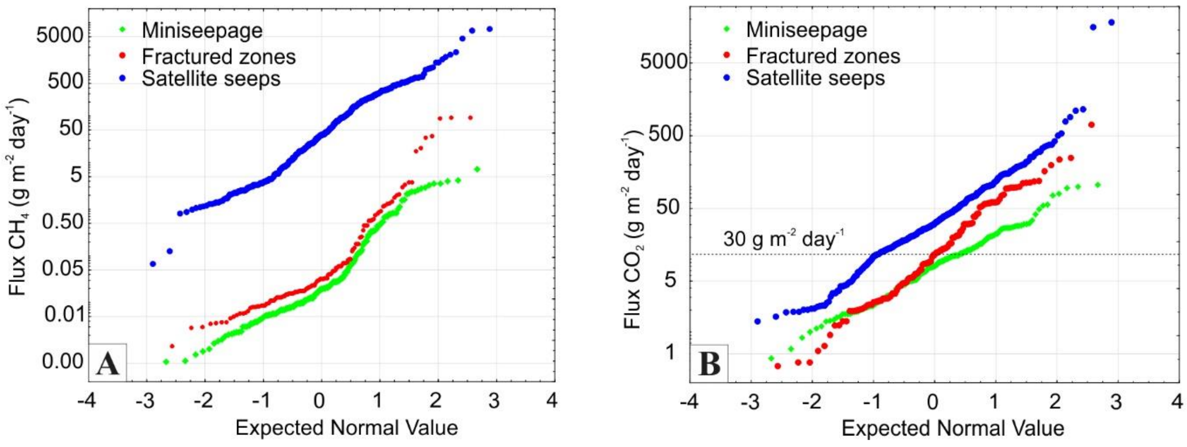

Fig. S1. *(A-B) Normal probability plots for* CH_4 *and* CO_2 *to define the background values and the anomalous populations. Note logarithmic scale of ordinate (y) axis.*

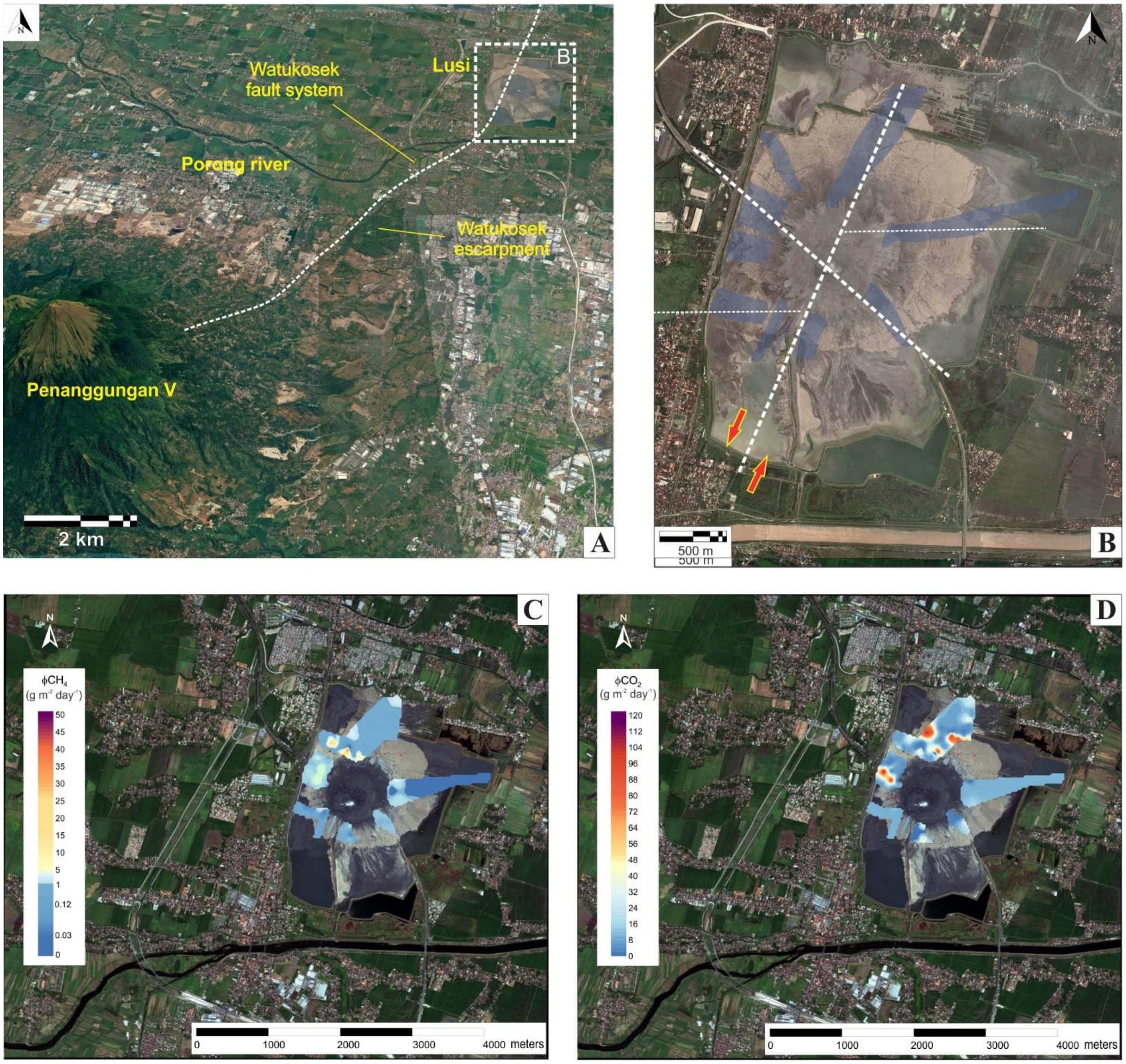

Fig. S2. *Fractured zones inside the embankment area. A) Google Earth satellite image of the region around Lusi. Indicated are the main features (moving towards the NE: Penanggungan volcano of the Arjuno-Welirang volcanic complex, the fault outcrop of the Watukosek escarpment, the bent path of the Porong River and the intersection with Lusi); (B) High resolution Ikonos satellite image and details of the fractured and faulted area inside the Lusi embankment based on field and satellite observations. Grey shaded areas indicate the zones with high amount of fractures, dashed lines trace the direction of the main and the antithetic fractures on field; (C-D) elaboration maps of* CH_4 *and* CO_2 *flux from the faulted areas. This figure has been constructed using Surfer 12.0 (Golden Sofware).*

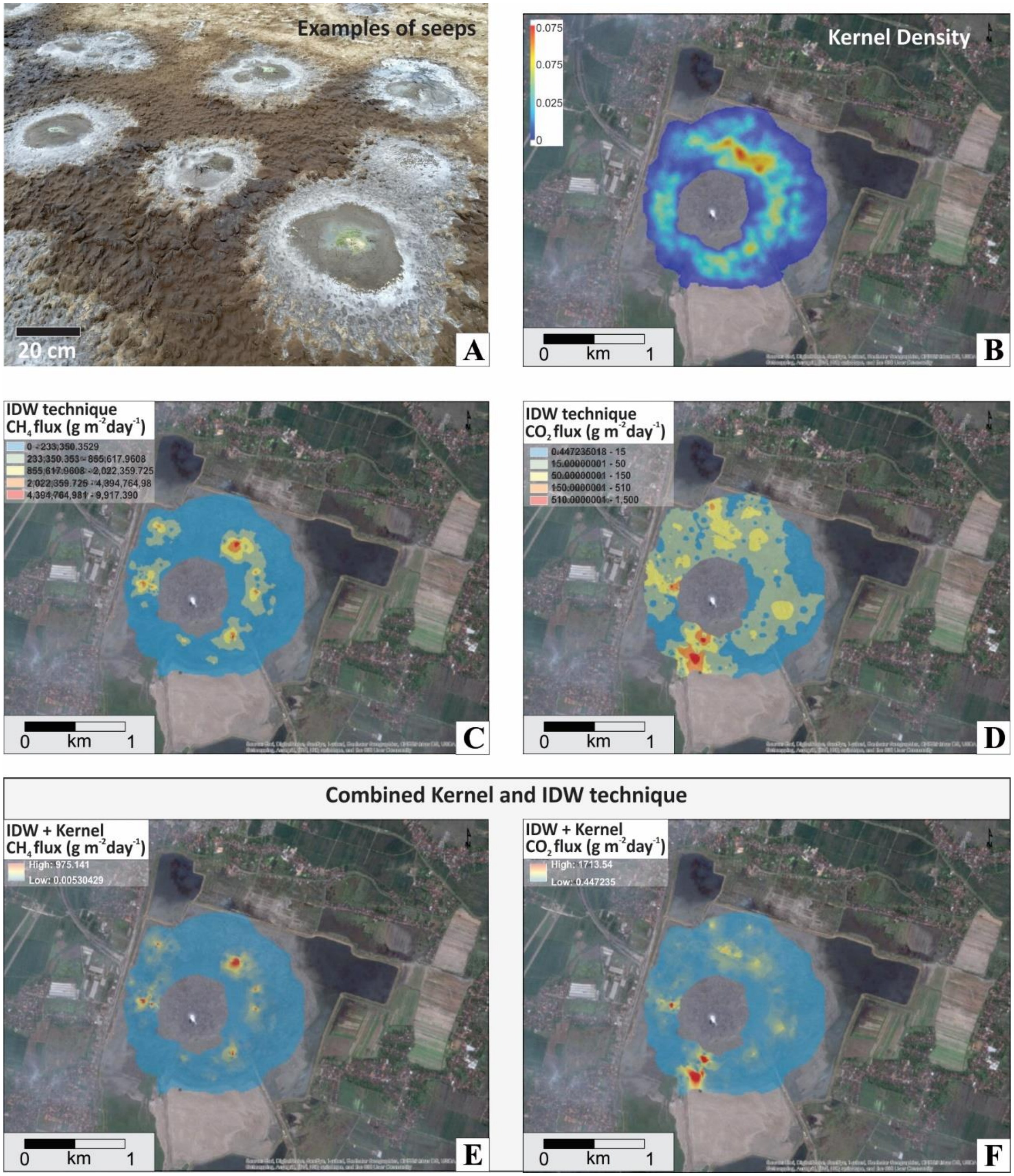

Fig. S3. *Satellite seeps inside the embankment area. A) Group of active seeps inside the embankment. (B) Superposed Ikonos satellite view and Kernel density; (C-D) Calculated* CH_4 *and* CO_2 *flux using IDW technique; (E-F) Combined Kernel and IDW technique for* CH_4 *and* CO_2 *fluxes. Basemap obtained using Statistica 10.0.*

2. Total emission estimates

Crater zone: The largest active vent (Vent 1) was monitored during its regular activity (i.e. excluding the powerful geysering events that characterize about half of the activity at Lusi; [11]). Between 2015-2019, a total of 205 replicated measurements were completed during low (< 1 m s^{-1}) wind conditions using a theodolite (Topcon AT-G2) positioned in the observatory tower located in the SE of the embankment zone (Fig. 1A, 2A). It was then measured the time required for a beamed point of the vapour plume of the main vent to move from a given height A to another height B. This large database shows that the speed of the rising plume ranges from 0.4 to 1.5 m/s (Fig. S4A). The resulting average speed of the plume is 0.86 m s^{-1} (± 0.19 m/s standard deviation). Based on comparative satellite and drone images we estimated that the main vent has a diameter of ~100 m. However, observations show that during the regular Lusi activity a) the plume does not occupy the full width of the vent but rather its central area (width of ~50 m, Fig. 1C) and b) the plume density is not homogeneous throughput its volume and contains air pockets. Our conservative estimates consider that the plume has air content ranging between 70-80% (Fig. S4B).

Monitoring of the smaller vent (Vent 2) shows that its behaviour is similar to that of the larger one. A diameter of 35 m was selected with the same parameters used for the main vent. These conservative parameters (i.e. 50 and 35 m wide plumes, 20-30% gas volume, regular vent activity) were then used for calculations. The crater gas composition was calculated from a large sample suite that we collected directly from the crater since 2006 in agreement with the values measured by Vanderkluysen et al.[4]. We know that 98 vol.% of Lusi plume is composed by aqueous vapour, while the remaining ~2 vol.% consists of CO_2 (respectively 1.49 vol.%) and CH_4 (0.51 vol.%)[3,4].

The total amount of gas released inside the gas plume has been estimated as follow:

$$G_i = \rho_i \left[\alpha_i \left(v \cdot S \right) \right]$$

Where G_i is the mass flux of a specific gas i (CO_2 or CH_4); ρ_i is the density of the gas; α_i is the concentration of this gas in the total volume flux of the plume; v is the velocity in the plume (0.86±0.19 m/s) of cross-sectional area S (1962 m^2 for main vent and 1017m^2 for smaller vent). The gas density can be estimated using molar volume of the ideal gas at 1 atm and 40º C (V_m=25.696 l/mol) and molar mass (M_{CO2}=44 g/mol and M_{CH4}=16 g/mol) as $\rho_i = M_i/V_m$. The gas

concentration α_i is the product of gas concentration from the crater (0.01492 for CO_2 and 0.00508 for CH_4) and the concentration of crater gas within the air-polluted plume (0.25 ± 0.05). Using this method we estimate a total flux from the main vent of ~42 ktonnes CH_4 yr^{-1}, and ~ 339.5 ktonnes CO_2 yr^{-1} while the second vent emits ~ 21.8 ktonnes CH_4 yr^{-1}, and ~176 ktonnes CO_2 yr^{-1}.

Miniseepage and fracture zones: The data treatment of flux measurements on miniseepage and fractured zones involved the calculation of standard statistical parameters. The Sinclair method[12] was applied to define background values by means of normal probability plot (NPP). As suggested by Fig. S1A, the probability plots of ϕCH_4 (logarithmic positive values) confirm that flux values have a log-normal distribution and highlight the presence of two different populations both for miniseepage and fractured zones. Differently, most ϕCO_2 variations (Fig. S1B) can be ascribed to a single population, with the exception of the background value (30 g m^{-2} day^{-1}) and some outliers with $\phi CO_2 \geq 200$ g m^{-2} day^{-1}, likely related to the occurrence of fractures causing an increased soil permeability at a local scale.

Moreover, in order to estimate a background value of the regional flux and its expected gradual decrease furthering from the crater zone, several profiles have been extended up to 2.7 km outside the embankment zone (Fig. 2A). The CO_2 weighted average was calculated taking into account only the measurements above the background values (30 g m^{-2} day^{-1}), likely dominated by biological soil respiration, while for CH_4 all positive measured values were considered (Fig. S1A-B).

Miniseepage and fractured zones statistical elaboration allows to estimate the gas emission rates (expressed in tonnes day^{-1}) by Natural Neighbour interpolation and volume method. In particular, a total of 175 flux measurements were carried out to estimate the miniseepage emission type. At any of these localities flux of CO_2 and CH_4 was detected resulting in mean values of 13.07 g m^{-2} day^{-1} (ϕCO_2) and 361 mg m^{-2} day^{-1} (ϕCH_4). This value has been applied for all the area framed by the embankment interested by miniseepage (5.91 km^2) resulting in a total average flux of 2.01 (from 1.3 to 4.8 lower and upper quartile, respectively) tonnes day^{-1} of CO_2 and 2.12 (from 0.9 to 5.6 lower and upper quartile, respectively) tonnes day^{-1} of CH_4.

The 129 flux measurements completed in the fractured regions (Fig. S2) revealed a CO_2-dominated seeping gas and have significantly higher soil temperatures and flux rates compared with the

surrounding undisturbed sediments. The calculated mean values are 35.75 g m^{-2} day^{-1} and 3.22 mg m^{-2} day^{-1} for ϕCO_2 and ϕCH_4, respectively. These values have been applied for the mapped fractured zones (1.34 km^2) resulting in a total average flux of 47.90 (from 15 to 100 lower and upper quartile, respectively) tonnes day^{-1} of CO_2 and 4.31 (from 0.04 to 19 lower and upper quartile, respectively) tonnes day^{-1} of CH_4. Fractured zones also show high concentration of active seeps confirming a greater amount of fluids release at these localities.

Satellite seeps: The average calculated flux rate among the 351 measured seeps is 71.68 g m^{-2} day^{-1} of CO_2 and 218.31 g m^{-2} day^{-1} of CH_4. The amount of CH_4 and CO_2 released by the satellite seeps was obtained by adding together the individual contribution of each measured seep.

Since the amount of satellite seeps occurring inside the embankment largely exceed the amount of those measured, the position of all the seeps was digitized based on high resolution satellite images. Their location can be easily recognized due to the colour contrast between the dry and the water-saturated sediments (Fig. S3A). Normal probability plots were used to calculate the population classes of the measured fluxes. The emission factor for CO_2 and CH_4 is the result of the weighted average for every single population class and the mean of the weighted averages of all the identified classes. These emission factors were applied for the number of seeps (total of 16,148) that have been mapped from the high resolution satellite images (Fig. 1A). Moreover, an additional statistical procedure was then applied in order to estimate the amount of CO_2 and CH_4 outgassing from these digitized seeps (i.e. defined as "points" in the following) but not measured in situ. The procedure combines the following statistical processing: *i)* Natural Neighbour that interpolates a raster surface from points using a natural neighbour technique; *ii)* IDW that interpolates a raster surface from points using an inverse distance weighted (IDW) technique; *iii)* Kernel Density that calculates a magnitude per unit area from point or polyline features using a Kernel function to fit a smoothly tapered surface to each point or polyline; *iv)* Raster Calculator that builds and executes a single Map Algebra expression using Python syntax in a calculator-like interface.

More details and references about these processing procedures are provided below. Natural Neighbour technique is a common and robust interpolation method that produces a conservative and artifice-free result. This approach aims to find, among the samples, the closest subset to each interpolation point and then applies weighted averages on them based on proportionate areas to

interpolate a value[13-16]. The interpolated surface is tightly controlled by the original data points and has the advantage of not having to specify parameters such as radius, number of neighbours or weights. The use of this method is most appropriate where sample data points are distributed with uneven density. The Inverse Distance Weighted (IDW) technique is an interpolation method usually applied to highly variable data assuming that each input point (i.e., digitized filtrations) has a local influence that decreases with distance. The use of this method assumes that the closer points have a greater weight than those further away and therefore their influence decreases with the distance from their sampled position[17-21]. Kernel density estimation is a statistical tool that allows to create a smooth curve given a set of data and to visualize the "shape" of some data, as a kind of continuous replacement for the discrete histogram[22-24]. Conceptually, a smoothly curved surface is fitted over each point. The surface value is highest at the location of an analysed point and diminishes with increasing distance ultimately reaching zero at the search radius distance (defined as 2 m based on concentric measurements conducted around a set of reference seeps) from the point. The digitalized seeps were analyzed and integrated using Raster Calculator to estimate and map their distribution in the study area[25-28].

The final product results from the merged Kernel and IDW maps (Fig.S3 B-F). A total flux of 14.59 and 98.52 tonnes day^{-1} of CO_2 and CH_4, respectively, was therefore calculated for all the seeps within the embankment.

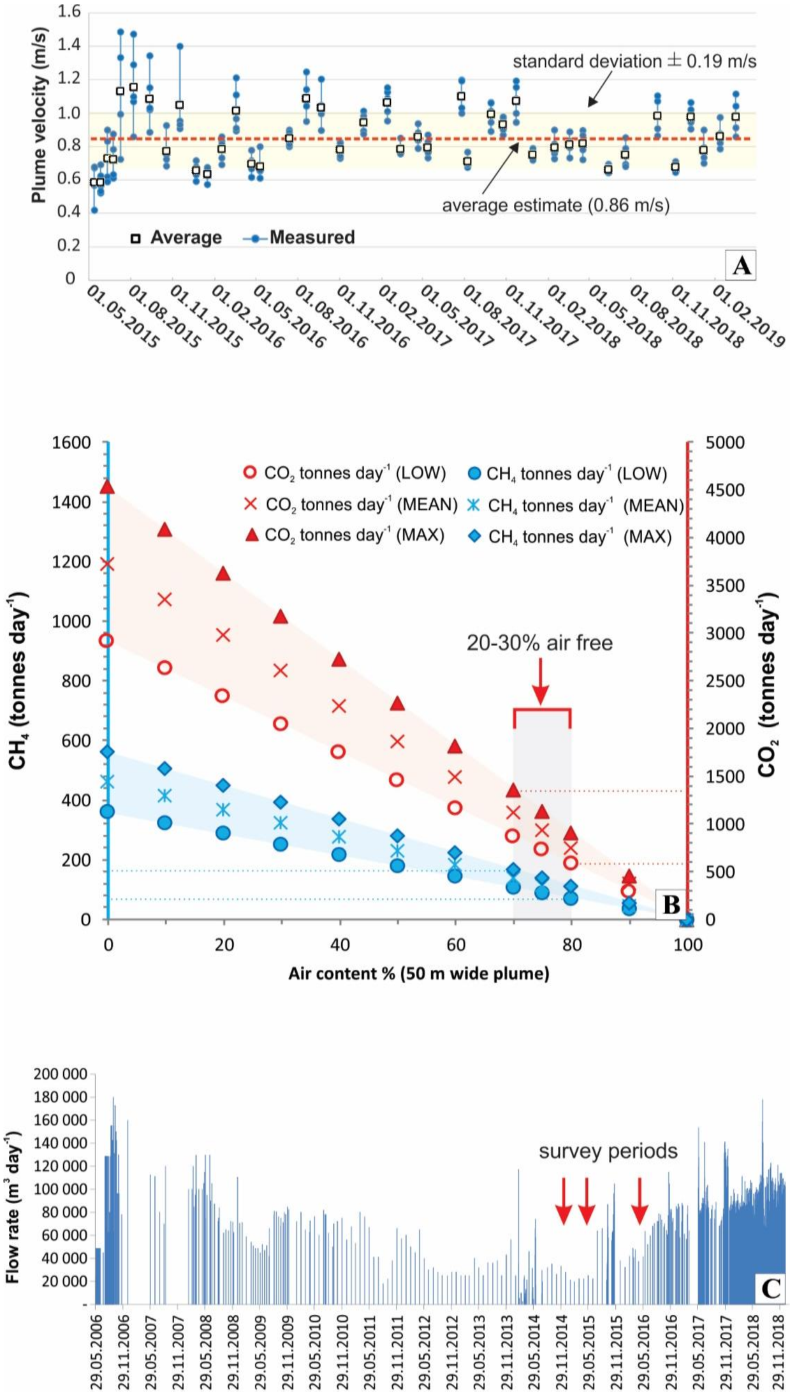

Fig. S4. *(A) Measured plume velocities since May 2015-March 2019. Indicated with the red line the average value calculated from the 205 measurements; shaded area indicates the standard deviation used for calculations. (B) Plot of daily* CO_2 *and* CH_4 *discharge from a 50 m wide plume during its regular activity. Considering a conservative gas content in the plume (i.e. between 20-30%) the daily flux is estimated to vary between 583-1358 tonnes day*$^{-1}$ *of* CO_2 *and 72-168 tonnes day*$^{-1}$ *of* CH_4*. (C) Mud flow rate measured since Lusi inception.*

3. Mud flow rate

Since the Lusi inception in May 2006, different approaches have been used to estimate the mud flow rate (Fig. S4C). Some of these methods are linked to the continuously mutating field conditions, the size of the eruption site, and the contingency procedures required to mitigate potential hazardous situations. These field operations were either combined or modified through time and had to be all taken into account in order to obtain the total flow rate (see more details in [29]). During the initial phases, the mud flow extension was monitored using satellite images and a portion of the erupted mud was scooped and loaded into large trucks so it could be removed, treated, and dumped at selected locations. In parallel several framed ponds were built around the crater zone to protect the settlements and control the spreading of the mudflows. The surface of these ponds was calculated using multiple IKONOS satellite images as well as measuring their empty volume. The flow rate within these ponds was then monitored using calibrated sticks. Since 2006 the region has been constantly monitored by a dense GPS network in order to measure potential subsidence in the area, and the flow rate estimates were compensated with recorded vertical movements[29]. Since October 2006, the erupted mud was treated and discharged, through a system of pumps, to the Porong River located to the south and later, in much lesser volumes, also to the stream located to the north of Lusi. Since March 2009 the active vent remained isolated at the centre of the large embankment zone and the erupted mud was forced towards the south to optimize the flushing into the Prong River. In order to implement the removal of clasts and fine-grained sediments deposited in the outskirts of the embankment zone, a "circular" pumping system strategy was applied when needed. First, water from the Porong River was pumped inside the outermost sectors of the embankment area; these newly created pools were then dredged in order to stir up in suspension the solid particles and ultimately pump all the sediment-enriched mixture out into the Porong River. The flow rate calculations were compensated with the amount of fresh water pumped inside these pools. All the estimates were compensated by the measured precipitations. Considering the significant amount of water evaporation that occurs directly at the crater site, in the surrounding large pond, and in the remaining area inside the embankment, the flow estimates might represent a lower value than the one reported herein.

4. TROPOMI - Emission quantification using the source pixel method

The TROPOMI instrument onboard the European Space Agency's Sentinal-5P satellite was launched in October 2017. The satellite is in a sun-synchronous orbit with a local overpass time of 13:30 hours. It has a wide swath of 2600 km and provides column averaged CH_4 mixing ratios (XCH_4), among other species, at ~7×7 km^2 ground pixel resolution at nadir and near daily global coverage. We analyzed data collected between May 2018 and July 2019 for local enhancements in XCH_4 over Lusi to provide an independent quantification of methane emissions and compare it with the estimates derived from in-situ measurements.

We use the mass balance method, described in Buchwitz et al.[30], to quantify the methane emission from Lusi. The mass balance method has the advantage that it is a quick method and does not require an atmospheric transport model. However, the method makes simplistic assumptions about the CH_4 emission distribution and atmospheric transport. In this method, the source flux Q [Gg y^{-1}] is calculated by multiplying the ΔXCH_4 enhancement (XCH_4 *source* – XCH_4 *background*) with a conversion factor CF shown below:,

$$Q = \Delta XCH_4 \times CF$$

$$CF = L \times V \times M_{exp} \times M \times C$$

where L *[km]* is the effective length of the source area through which the air parcel carrying methane is ventilated (computed as square root of the source region), V *[km h^{-1}]* is the effective wind speed, M_{exp} is the ratio of average surface pressure in the source box and standard surface pressure of 1013.25 hPa, M is a constant to convert concentration to mass change per area *[5.345 kg CH_4 km^{-2} ppb^{-1}]* in standard atmospheric conditions and C is a dimensionless factor chosen to be 2.0, derived by Buchwitz et al.[30], based on the concentration difference of the air parcel before and after entering the source area.

The domain of the TROPOMI analysis ranges from 6.027 to 9.027 °S latitude and 111.211 to 114.211 °E longitude (Fig. S5).

The source box 1 (see Fig. S5) covers a rectangular area of ~20×20 km^2 centered around the Lusi eruption site located at 112.711 ^oE, 7.527 ^oS. To calculate the XCH_4 enhancement in the source box, the local background mixing ratio in the upwind direction of the source pixels is subtracted. The local background region is chosen in upwind direction of the source box, with the dimension equal to the length of the source box, which lies to the East and South-East of Lusi given the

dominant wind direction during the analyzed period (see Fig S6A). The spatial distribution of XCH_4 is corrected for the influence of variations in surface elevation by adding 7 ppb/km relative to mean sea level[30]. Boundary layer averaged wind fields from ECMWF ERA5 are used, representing the 06:00 UTC overpass time of TROPOMI (C3S, 2017). Figure S6B shows the wind speed and direction for each grid pixel of ECMWF ERA5 winds over the domain from all the considerable days of the screened orbits. Since the selected source box is larger than the area of Lusi (~7 km^2), other sources may be present. To account for their contribution, anthropogenic emissions from EDGAR v5.0 for 2015;[31], biomass burning emissions from the GFED4.1s emission inventory for 2018;[32] and wetland emissions from WetCHARTs version 1.0;[33] for 2015 were subtracted. Emissions were quantified for boxes 1-6 (Fig. S5), with varying contributions from anthropogenic emissions to test the robustness of the emission estimate for Lusi.

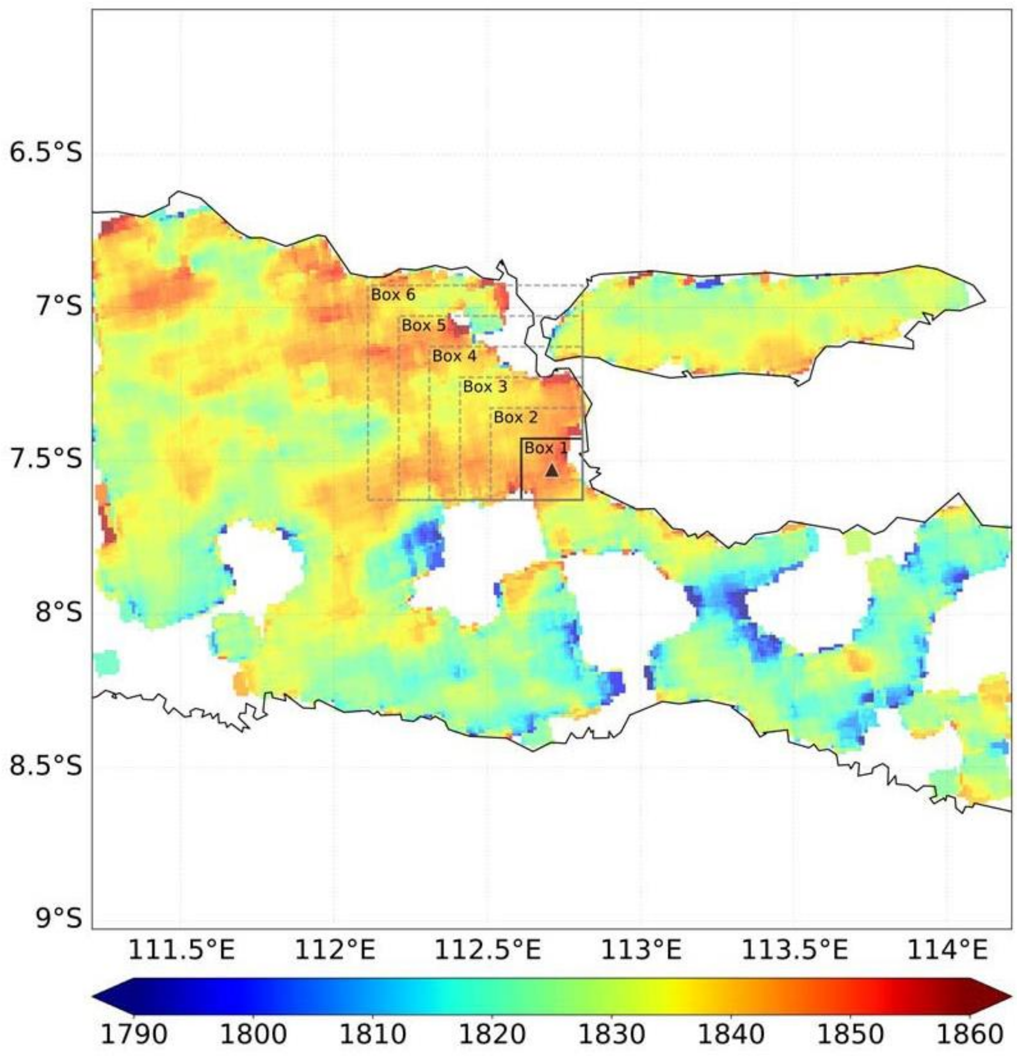

Fig. S5. *An oversampled (0.01° × 0.01°) map of TROPOMI data averaged over the study domain from May 2018 till July 2019. The location of Lusi is indicated by a triangle and the squares denotes the multiple source boxes considered for emission quantification. Units in ppb. Map generated using Python 2.7.13 version.*

Case for sensitivity: Data averaging and bias correction

A total of 50 orbits were screened for data availability, in the period May 2018 – July 2019 requiring > 100 pixels per orbit, to have sufficient spatial coverage over the domain. Figure S6C show the temporal spread of screened orbits across the analysis period. The data in these orbits

were regridded and averaged at a resolution of 0.1° × 0.1°. The selected orbits show a significant correlation between XCH_4 and aerosol optical thickness (AOT) varying from -0.13 to -0.86, with a correlation of -0.68 averaged over all data. The averaged data were corrected to remove this correlation by linear regression between XCH_4 and AOT (Fig. S6D), yielding a slope of -364 ppb per unit of AOT. As a sensitivity test, emissions were quantified using TROPOMI data including and excluding this additional bias correction.

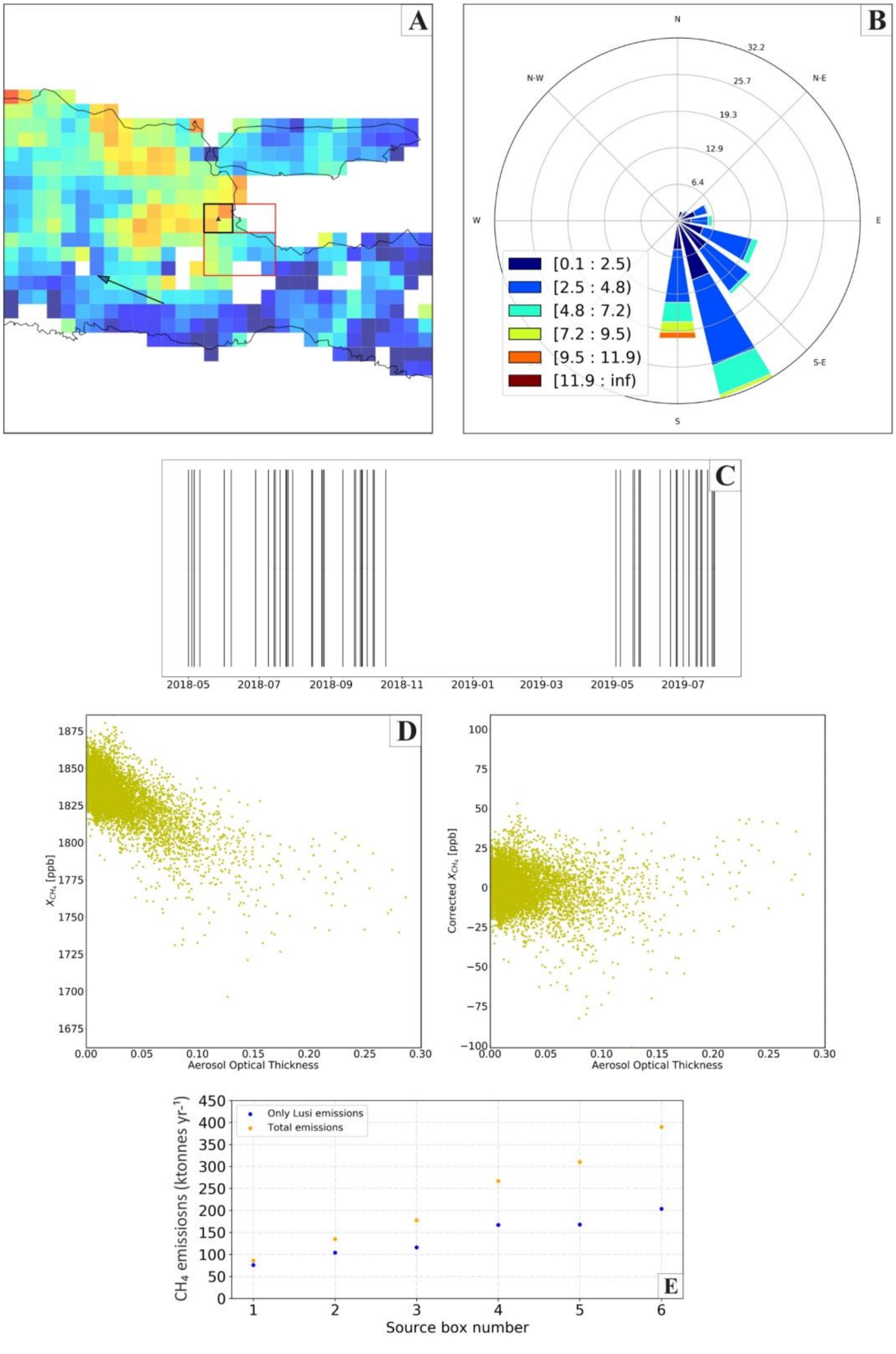
A
B
N
N-W
N-E
W
E
S-E
S
32.2
25.7
19.3
12.9
6.4
[0.1 : 2.5)
[2.5 : 4.8)
[4.8 : 7.2)
[7.2 : 9.5)
[9.5 : 11.9)
[11.9 : inf)
C
2018-05
2018-07
2018-09
2018-11
2019-01
2019-03
2019-05
2019-07
D
X_{CH_4} [ppb]
Corrected X_{CH_4} [ppb]
Aerosol Optical Thickness
Aerosol Optical Thickness
E
Only Lusi emissions
Total emissions
CH_4 emissiosns (ktonnes yr-1)
Source box number

Fig. S6. *Mass balance method defining regions, bias correction and final estimates for TROPOMI. (A) Local background region (red polygon) as defined in the upwind direction of source box (black polygon) for TROPOMI measurements. The dominant wind direction is arrowed. (B) Windrose diagram showing dominant wind speed and wind direction from all the grids of considered orbits (domain: 112.711 ± 0.525, -7.527 ± 0.525). (C) Screened orbits considered for the analysis spread across different months in 2018-2019. On the x-axis we have the temporal scale and each vertical line along y-axis represents screened orbit considered for the analysis. (D) The relation between TROPOMI* XCH_4 *and AOT before (left panel) and after (right panel) correction using linear regression. (E) Methane emissions quantified from TROPOMI data using source boxes increasing in size (see Fig. S5 for the definition of box 1-6). Total emissions include emissions from Lusi, EDGARv5.0 anthropogenic, GFED4.1s biomass burning and wetlands. All images were generated using Python 2.7.13 version.*

CH_4 emission from Lusi

In this study, we use the official TROPOMI operational two-band retrieval product. It uses the O_2A band at 0.79 μm and the CH_4 band at 2.3 μm in respectively the NIR and SWIR spectral bands of TROPOMI. XCH_4 is retrieved using the full-physics RemoTeC algorithm, accounting for light path perturbations due to scattering on aerosol and cirrus cloud particles in the atmosphere[34,35]. Butz et al.,[35] showed that XCH_4 from the full-physics retrieval algorithm used for TROPOMI data can have large errors over dark surfaces. This can affect our emissions estimates. Therefore, we filter out TROPOMI pixels with SWIR albedo of less than 0.02 (ATBD [36]). Using the mass balance approach outlined above, the CH_4 emission from Lusi was estimated at 140 ± 87 ktonnes yr^{-1}. This number is consistent with the independent estimate that is obtained from surface data, albeit that uncertainty margins are large. The sensitivity test using corrected data gave an estimate of 70 ktonnes yr^{-1}, which lies within the high uncertainty range of our estimate. Using the ensemble of estimates due to varying background, source box and regridding resolution we calculate an uncertainty of 60% around the quantified mean emission. For the wind speed, we estimate an uncertainty of 18% (around the mean) which is similar to that found in measurements i.e. 22% (0.19/0.86 m s^{-1}) (Figure S4A). Moreover, the surface data are expected to lead to a lower emission rate due to a low mud flow rate during the 2014-2016 in situ survey period (Fig S4C). The TROPOMI measurements where conducted for the period 2018-2019. During this time interval

mud flow rates were higher, with likely increased fluxes of water vapor, CO_2 and CH_4 (Fig. S4C). The high uncertainty in the TROPOMI-derived estimate is mainly due to the sensitivity to the choice of the background and source boxes. The anthropogenic emission from EDGARv5.0 varies between 10 ktonnes yr^{-1} and 184 ktonnes yr^{-1} while that of GFED4.1s varies between 0.09 to 0.41 ktonnes yr^{-1} and wetland varies from 0.04 to 1.65 ktonnes yr^{-1} going from ~20×20 km^2 to ~70×70 km^2, and therefore become significant at larger source box sizes. Further details about the estimates and other parameters are tabulated in Table S1. Our emission quantification method cannot clearly distinguish all sources of emissions in the vast region around Lusi. These could include unusually high anthropogenic sources (not identified so far on the basis of the information we got from maps and local authorities) and additional natural geological emissions such as the seepage activity that has been reported in the region (e.g. [37 and refs therein]). In any case, the TROPOMI estimates are consistent with a significant source from Lusi for any choice of region (Fig. S6E).

Source region	Size (deg)	Dimension (Lat-Long) (deg)	Wind Speed (m/s)	Total mass balance (Gg/y)	EDGAR v5.0 (Gg/y)	GFED v4.1s (Gg/y)	Wetland (LPJ) (Gg/y)	Lusi (Gg/y)
Box 0	0.2° × 0.2°	(-7.427 : -7.627) (112.611 : 112.811)	1.36 ± 0.12	86	10.02	0.09	0.04	76 ± 24
Box 1	0.3° × 0.3°	(-7.327 : -7.627) (112.511 : 112.811)	1.52 ± 0.15	135	31.09	0.19	0.13	104 ± 30
Box 2	0.4° × 0.4°	(-7.227 : -7.627) (112.411 : 112.811)	1.42 ± 0.13	178	61.58	0.27	0.17	116 ± 33
Box 3	0.5° × 0.5°	(-7.127 : -7.627) (112.311 : 112.811)	1.76 ± 0.08	267	99.54	0.33	0.45	167 ± 61
Box 4	0.6° × 0.6°	(-6.977 : -7.627) (112.211 : 112.811)	1.56 ± 0.05	310	140.98	0.37	0.98	168 ± 56
Box 5	0.7° × 0.7°	(-6.927 : -7.627) (112.111 : 112.811)	1.87 ± 0.08	390	184.15	0.41	1.65	204 ± 68

Table S1: *Input parameters used to calculate methane emissions at Lusi eruption site for different source input.*

References

1. Procesi, M., Ciotoli, G., Mazzini, A. & Etiope, G. Sediment-hosted geothermal systems: Review and first global mapping. *Earth-Science Reviews* **192**, 529-544, (2019).
2. Mazzini, A. & Etiope, G. Mud volcanism: An updated review. *Earth-Science Reviews* **168**, 81–112, (2017).
3. Mazzini, A., Etiope, G. & Svensen, H. A new hydrothermal scenario for the 2006 Lusi eruption, Indonesia. Insights from gas geochemistry. *Earth and Planetary Science Letters* **317**, 305-318, (2012).
4. Vanderkluysen, L., Burton, M. R., Clarke, A. B., Hartnett, H. E. & Smekens, J. F. Composition and flux of explosive gas release at LUSI mud volcano (East Java, Indonesia). *Geochemistry Geophysics Geosystems* **15**, 2932-2946, (2014).
5. Di Felice, F., Mazzini, A., Di Stefano, G. & Romeo, G. Drone high resolution infrared imaging of the Lusi mud eruption. *Marine and Petroleum Geology* **90**, 38-51, (2018).
6. Etiope, G. Natural Gas Seepage. The Earth's hydrocarbon degassing. *Springer International Publishing Switzerland, ISBN 978-3-319-14601-0 (eBook), DOI 10.1007/978-3-319-14601-0.*, 199, (2015).
7. Mazzini, A. *et al.* Strike-slip faulting as a trigger mechanism for overpressure release through piercement structures. Implications for the Lusi mud volcano, Indonesia. *Marine and Petroleum Geology* **26**, 1751-1765, (2009).
8. Sciarra, A. *et al.* Radon and carbon gas anomalies along the Watukosek Fault System and Lusi mud eruption, Indonesia. *Marine and Petroleum Geology* **90**, 77-90, (2018).
9. Mauri, G. *et al.* Insights on the structure of Lusi mud edifice from land gravity data. *Marine and Petroleum Geology* **90**, 104-115, (2018).
10. Moscariello, A. *et al.* Genesis and evolution of the Watukosek fault system in the Lusi area (East Java). *Marine and Petroleum Geology* **90**, 125-137, (2018).
11. Karyono, K. *et al.* Lusi, a clastic-dominated geysering system in Indonesia recently explored by surface and subsurface observations. *Terra Nova* **29**, 13-19, (2017).
12. Sinclair, A. J. Selection of threshold values in geochemical data using probability graphs. *J. Geochem. Explor.* **3**, 129–149, (1974).
13. Watson, D. F. & Philip, G. M. Neighborhood-based interpolation. *Geobyte* **2**, 12–16, (1987).
14. Sibson, R. in *Interpreting Multivariate Data:* (ed V. Barnett) 21–36 (John Wiley & Sons, 1981).
15. Sambridge, M., Braun, J. & McQueen, H. Geophysical parameterization and interpolation of irregular data using natural neighbours. *Geophys. J. Int.* **122**, 837–857., (1995).
16. Bobach, T., Farin, G., Hansford, D. & Umlauf, G. Natural neighbor extrapolation using ghost points. *Computer aided design* **41**, 350-365, (2009).
17. Bartier, P. M. & Keller, C. P. Multivariate interpolation to incorporate thematic surface data using inverse distance weighting (IDW). *Computers & geosciences* **22**, 795-799, (1996).
18. Mueller, T. G. *et al.* Map Quality for Ordinary Kriging and Inverse Distance Weighted Interpolation. *Soil Science Society of America journal* **68**, 2042-2047, (2004).
19. Yang, W.-R., Wang, R.-S., Huang, J.-L., Chen, Z. & Li, F. Application of inverse distance weighted interpolation method in contaminated site assessment. *Ying Yong Sheng Tai Xue Bao* **18**, 2013, (2007).

20 Watson, D. F. & Philip, G. M. A refinement of inverse distance weighted interpolation. *Geo-Processing*, **2**, 315-327, (1985).

21 Setianto, A. & Triandini, T. Comparison of kriging and inverse distanceweighted (IDW) interpolation methods in lineament extraction and analyses. *Journal of Applied Geology* **5**, (2015).

22 Silverman, B. W. *Density estimation for statistics and data analysis*. (Chapman and Hall, 1986).

23 Scott, D. W. *Multivariate density estimation : theory, practice, and visualization*. (Wiley, 1992).

24 Węglarczyk, S. Kernel density estimation and its application. *ITM Web of Conferences 23, 00037 (2018) XLVIII Seminar of Applied Mathematics, https://doi.org/10.1051/itmconf/20182300037*, (2018).

25 Schintler, L. A., Kulkarni, R., Gorman, S. & Stough, R. Using Raster-Based GIS and Graph Theory to Analyze Complex Networks. *Networks and spatial economics* **7**, 301-313, (2007).

26 Gelagay, H. S. & Minale, A. S. Soil loss estimation using GIS and Remote sensing techniques: A case of Koga watershed, Northwestern Ethiopia. *International Soil and Water Conservation Research* **4**, 126-136, (2016).

27 Wang, Y. Y., Wen, A. B., Guo, J., Shi, Z. L. & Yan, D. C. Spatial distribution, sources and ecological risk assessment of heavy metals in Shenjia River watershed of the Three Gorges Reservoir Area. *Journal of Mountain Science* **14**, 325-335, (2017).

28 Habel, M. *et al.* Using the raster calculator tool to appraise riverbed elevation changes nearby hydrotechnical objects on alluvial rivers. Monitoring 2019. *Published by European Association of Geoscientists & Engineers. https://doi.org/10.3997/2214-4609.201903230* **2019**, 1-5, (2019).

29 Istadi, B. P., Pramono, G. H., Sumintadireja, P. & Alam, S. Modeling study of growth and potential geohazard for LUSI mud volcano: East Java, Indonesia. *Marine and Petroleum Geology* **26**, 1724-1739, (2009).

30 Buchwitz, M. *et al.* Satellite-derived methane hotspot emission estimates using a fast data-driven method. *Atmos Chem Phys* **17**, 5751-5774, (2017).

31 Crippa, M. *et al.* Fossil CO2 and GHG emissions of all world countries: 2019 report. *Report, EUR 29849 EN, Publications Office of the European Union, Luxembourg, 2019, ISBN 978-92-76-11100-9, doi:10.2760/687800, JRC117610*, (2019).

32 van der Werf, G. R. *et al.* Global fire emissions estimates during 1997-2016. *Earth Syst Sci Data* **9**, 697-720, (2017).

33 Bloom, A. A. *et al.* A global wetland methane emissions and uncertainty dataset for atmospheric chemical transport models (WetCHARTs version 1.0). *Geosci. Model Dev.* **10**, 2141-2156, (2017).

34 Hu, H. L. *et al.* The operational methane retrieval algorithm for TROPOMI. *Atmos Meas Tech* **9**, 5423-5440, (2016).

35 Butz, A. *et al.* TROPOMI aboard Sentinel-5 Precursor: Prospective performance of CH4 retrievals for aerosol and cirrus loaded atmospheres. *Remote sensing of environment* **120**, 267-276, (2012).

36 Hasekamp, O. *et al.* Algorithm Theoretical Baseline Document for Sentinel-5 Precursor methane retrieval. *http://www.tropomi.eu/documents/atbd/* **SRON-S5P-LEV2-RP-001**, (2019).

37 Zaputlyaeva, A., Mazzini, A., Caracausi, A. & Sciarra, A. Mantle-Derived Fluids in the East Java Sedimentary Basin, Indonesia. *Journal of Geophysical Research: Solid Earth* **124**, 7962-7977, (2019).
